# Effect of probiotics or prebiotics on thyroid function: A meta-analysis of eight randomized controlled trials

**DOI:** 10.1371/journal.pone.0296733

**Published:** 2024-01-11

**Authors:** Qinxi Shu, Chao Kang, Jiaxin Li, Zhenzhu Hou, Minfen Xiong, Xingang Wang, Hongyan Peng

**Affiliations:** 1 Hospital of Chengdu University of Traditional Chinese Medicine, Chengdu, Sichuan Province, China; 2 Chengdu University of Traditional Chinese Medicine, Chengdu, Sichuan Province, China; 3 Department of Nutriology of the General Hospital of Western Theater Command, Chengdu, Sichuan Province, China; 4 Department of Emergency Medicine, No. 922 Hospital of the Joint Service Support Force of the PLA, Hengyang, Hunan Province, China; 5 Department of Critical Care Medicine, No. 922 Hospital of the Joint Service Support Force of the PLA, Hengyang, Hunan Province, China; 6 Department of Health Medicine, No. 922 Hospital of the Joint Service Support Force of the PLA, Hengyang, Hunan Province, China; Universidad San Francisco de Quito, ECUADOR

## Abstract

**Background:**

Microbiome-directed therapies are increasingly utilized to optimize thyroid function in both healthy individuals and those with thyroid disorders. However, recent doubts have been raised regarding the efficacy of probiotics, prebiotics, and synbiotics in improving thyroid function. This systematic review aimed to investigate the potential relationship between probiotics/prebiotics and thyroid function by analyzing the impact on thyroid hormone levels.

**Methods:**

We conducted a comprehensive systematic review and meta-analysis of randomized controlled trials that investigated the effects of probiotics, prebiotics, and synbiotics on free triiodothyronine (fT3), free thyroxine (fT4), thyroid stimulating hormone (TSH), and thyroid stimulating hormone receptor antibody (TRAb) levels. We searched for articles from PubMed, Scopus, Web of Science, and Embase up until April 1st, 2023, without any language restriction. Quantitative data analysis was performed using a random-effects model, with standardized mean difference (SMD) and 95% confidence interval as summary statistics. The methods and results were reported according to the PRISMA2020 statement.

**Results:**

A total of eight articles were included in this review. The meta-analysis showed no significant alterations in TSH (SMD: -0.01, 95% CI: −0.21, 0.20, *P* = 0.93; I^2^: 0.00%), fT4 (SMD: 0.04, 95% CI: −0.29, 0.21, *P* = 0.73; I^2^: 0.00%) or fT3 (SMD: 0.45, 95% CI: −0.14, 1.03, *P* = 0.43; I^2^: 78.00%), while a significant reduction in TRAb levels was observed (SMD: -0.85, 95% CI: -1.54, -0.15, *P* = 0.02; I^2^: 18.00%) following probiotics/prebiotics supplementation. No indication of publication bias was found.

**Conclusions:**

Probiotics/prebiotics supplementation does not influence thyroid hormone levels, but may modestly reduce TRAb levels in patients with Graves’ disease.

## 1 Introduction

Thyroid disease is becoming a global health problem that substantially impact people all over the world. It encompasses various conditions such as thyroid enlargement (nodules or goiter), hypothyroidism, hyperthyroidism, subclinical hypothyroidism, subclinical hyperthyroidism, structural abnormalities, and thyroid cancer [1]. Hypothyroidism is characterized by thyroid-stimulating hormone (TSH) levels above the upper limit of the reference range while levels of free thyroxine (fT4) are below the lower limit of the reference range. In the United States, overt and subclinical hypothyroidism have prevalence rates of 0.4% and 9% respectively [2]. In Europe, the estimated annual incidence of hypothyroidism is 226 cases per 100,000 individuals [3]. Hyperthyroidism is also a common thyroid disorder with a global prevalence of 0.2–1.3%, predominantly caused by Graves disease (GD) [4]. It is diagnosed by biochemical tests, for example, low TSH, high fT4, or high free tri-iodothyonine (fT3) [5]. Thyroid cancer is the most common cancer of the endocrine system, accounting for 1–2% of all new cancers diagnosed each year worldwide, the incidence increased by 20 percent [6]. Epidemiological data indicate that several individual risk factors, including obesity and menopause, are associated with hypothyroidism (most commonly caused by Hashimoto thyroiditis (HT), and chronic autoimmune thyroid disease), subclinical hypothyroidism, and differentiated thyroid cancer (DTC) [7, 8]. A systematic review demonstrated a positive association between BMI and the risk of developing differentiated thyroid cancer (HR = 1.18, 95% CI, 1.03–1.35) [9]. Additionally, a large cross-sectional study reported that higher BMI was associated with an increased prevalence of thyroid cancer in women (OR = 1.63, 95% CI 1.24–2.10), indicating that BMI is a significant predictor of DTC in women [10]. Furthermore, a Chinese observational revealed a positive correlation between BMI and serum TSH levels in 1,816 men and 1,774 women, with postmenopausal individuals having lower TSH levels and a reduced risk of severe obesity [11].

The gut microbiota plays a pivotal role in thyroid disorders, including HT, GD and thyroid cancer. Recent data have suggested that microbes influence thyroid hormone levels through the regulation of iodine uptake, degradation, and enterohepatic cycling [12]. Studies have been shown that *Lactobacillaceae* and *Bifidobacteriaceae* are often reduced in hypothyroidism and hyperthyroidism [13]. In GD patients, the composition of the gut microbiota differs from that of healthy individuals, with a significant decrease in the relative abundance of *Faecalibacterium prausnitzii*, *Butyricimonas faecalis*, *Bifidobacterium adolescentis* and *Akkermansia muciniphila* compared to the control. Furthermore, a diagnostic model was developed using metagenome-assembled genomes of the gut microbiome, which may serve as a valuable predictor for GD [14]. The gut microbiota has the capacity to produce various neurotransmitters, such as dopamine, which can regulate the hypothalamus-pituitary axis (HPA) and inhibit TSH [15]. Numerous studies have been conducted to investigate the modulation of gut microbiota in order to restore dysbiosis in patients with thyroid disorders. Probiotics and prebiotics have demonstrated beneficial effects on thyroid diseases. Probiotics *bifidobacterium longum* supplied with methimazole was implemented in nine patients with GD for six months. The results showed a significant reduction in clinical thyroid indexes, including fT3, fT4, and thyrotropin receptor antibody (TRAb), while TSH levels increased compared to baseline [16]. Another study explored the effects of a four-week treatment with a complex probiotics preparation consisting of *Bifidobacterium infantis*, *Lactobacillus acidophilus*, *Enterococcus faecalis* and *Bacillus cereus* in patients post-thyroid hormone withdrawal (THW) following thyroid cancer surgery. The treatment led to a decreased occurrence of complications such as dyslipidemia and constipation. However, the serum levels of fT4, fT3 and TSH showed no change between groups [17]. Synbiotics, which are a mixture of probiotics and prebiotics, have also been examined [18]. They have shown potential in reducing TSH and increasing fT3 in patients with hypothyroidism [19]. A strategy based on microbiome-directed therapies, involving supplementation with probiotics, prebiotics and synbiotics holds promise as a therapeutic approach for thyroid disorders.

There is a growing interest in investigating the potential role of probiotics or prebiotics supplementation to improve thyroid function in humans. However, the results have been inconsistent, and the probiotics or prebiotics are various in each randomized clinical trial, leading a mixed effect. The primary objective of this study was to explore the effects of probiotics and prebiotics treatment on thyroid function, not limited to patients with thyroid disorders. To this end, an extensive literature search was performed in preprint platforms and databases, aiming to include all relevant trials in order to identify knowledge gaps and facilitate informed decision-making.

## 2 Methods

### 2.1 Search strategy

This systematic review was conducted following the guidelines outlined in the Preferred Reporting Items for Systematic Reviews and Meta-Analyses (PRISMA) statement and the Cochrane Handbook for Systematic Reviews [20]. The meta analysis was not registered before. A systematic search was performed in MEDLINE (via PubMed), Scopus (via Elsevier),Web of Science-Science Citation Index and Social Sciences Citation Index (via Clarivate) and Embase (via Elsevier) on April 1st, 2023, with no restrictions regarding language or year of publication. The search included the following keywords: “Thyroid diseases”, “Thyroid disorders”, “Thyroid function”, “hypothyroidism”, “hyperthyroidism”, “subclinical hypothyroidism”, “subclinical hyperthyroidism”, “thyroid cancer”, “Prebiotics”, “Probiotics”, “synbiotics”, “Yogurt”, “milk” and “Dairy product”. We also conducted a manual search on preprint platforms (medRxiv and Research Square) and other databases (CINAHL, China National Knowledge Internet databases, Wanfang Database). We manually searched gray literature through access to ClinicalTrials.gov, the Cochrane Central Registry of Controlled Trials, the Web of Science: Conference Proceedings Citation Index-Science (via Wiley) to identify completed but unpublished studies meeting our eligibility criteria to reduce publication bias. Authors were contacted to obtain missing data. Details of the systematic search terms are described in S1 and S2 Tables.

### 2.2 Eligibility criteria

We included studies on adult participants (18 to 65 years) that met the following criteria: (1) studies assessing the effects of probiotics or prebiotics treatment on thyroid functions in subjects with thyroid disease (eg. hypothyroidism, subclinical hypothyroidism, hyperthyroidism, subclinical hyperthyroidism and thyroid cancer), and those at risk of hypothyroidism, like obesity and menopause [21]. (2) The full text of the study was available. (3) The results were thyroid hormones (THs), including at least one of total triiodothyronine (TT3), fT3, total thyroxine (TT4), fT4 TSH and TRAb. (4) Studies were written in English. We excluded: (1) Studies on cell-level, animal, or model studies. (2) Letters, case reports, conference reports, laboratory studies, editorials, and any type of review. (3) Not randomized clinical trials (RCTs).

### 2.3 Data extraction

A standardized data collection sheet was designed and created in Microsoft Excel. One reviewer performed the initial data extraction for all included articles, while a second reviewer checked all proceedings. The following detailed information was collected from each included study: title, author, year, country, number of participants, age, sex, type and dose of probiotics or prebiotics, clinical characteristics of participants, TSH, fT4, TT4, fT3, TT3 and TRAb. In cases of missing information, the corresponding author was contacted via email to request the missing data.

### 2.4 Quality assessment

The risk of bias in the included studies was independently assessed by two investigators (QS, CK), and the results were cross-validated. Cochrane Risk of Bias tool 2.0 (ROB2) was employed for quality assessment [22]. The tool was used to assess as follows: randomization process, deviations from the intended intervention, outcome measurement, missing outcome data, and selection of reported results. Study quality was assessed by the Jadad scale with three items, including the randomization (0, 1 or 2), double blinding (0, 1 or 2) and withdrawals and dropouts (0 or 1). Studies receiving a score ≥4 were deemed to be of high quality, whereas those receiving a score <4 were considered lower quality [23]. In case of disagreements over the rating of a study, all authors examined the article and reached a consensus.

### 2.5 Statistical analysis

The information obtained from the included articles was combined using STATA v.17.0 software (College Station, TX: StataCorp LLC) and Review Manager v.5.4 (The Cochrane Collaboration, Copenhagen, Denmark). Random-effects model was chosen for all the meta-analysis. The between-study variance was estimated by DerSimonian and Laird method. The data from medians and interquartile ranges (IQR) were converted to means and standard deviations (SD) by using Hozo’s method [24]. For variables with the standard errors (SE) reported, SD was calculated by the following equation: SE × √ (sample size). The first or final time-point from each study in case of the results may be presented for several periods of follow-up. The between-study heterogeneity was evaluated using a chi-squared test and the I^2^ statistic. P-values < 0.1 were considered indicative of heterogeneity for the chi-squared test. Heterogeneity was classified as low if I^2^ < 30%, moderate if I^2^ = 30%-60%, and high if I^2^ > 60% [25]. Publication bias was assessed through funnel plot, Begg’s rank correlation and Egger’s weighted regression tests. When there was evidence of funnel plot asymmetry, potentially missing studies were imputed using the “trim and fill” method.

## 3 Results

### 3.1 Search results

**Fig 1:**
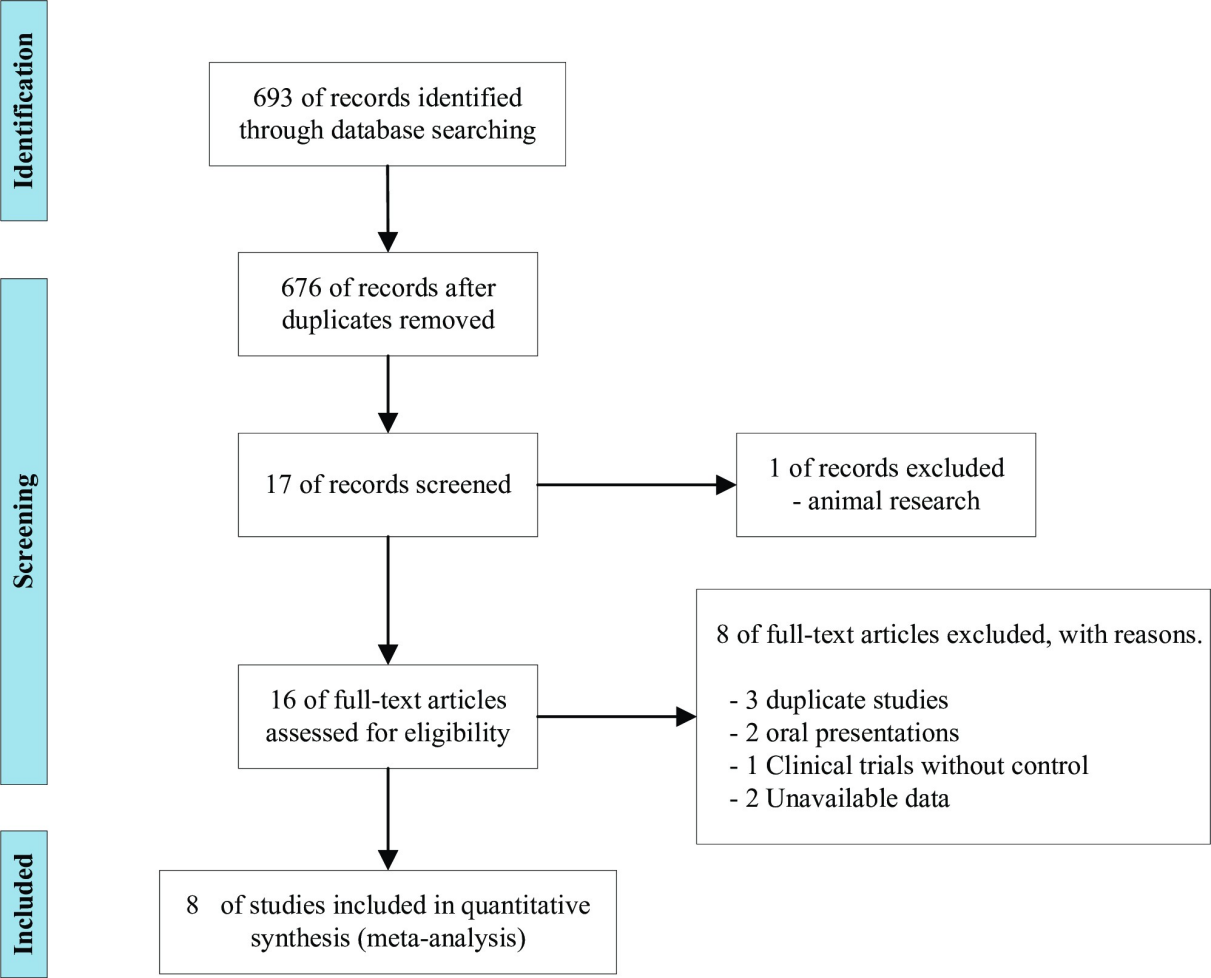
Flow diagram for identification of studies in the systematic review.

Our electronic search yielded a total of 693 articles. After evaluating the titles and abstracts of the studies, 676 papers were excluded and 16 articles remaining were retrieved for full-text screening and their eligibility was assessed for meta-analysis. Of these, three were duplicate research, two studies were oral presentations, where data on thyroid hormones were not provided, one was not designed for RCT, and two did not measure thyroid function. Finally, eight RCT studies were included in the systematic review and meta-analysis and they were all peer-reviewed publications (Fig 1).

### 3.2 Study characteristics

The eight studies included in this analysis were published between 2017 and 2023, and were conducted across three different nations (China, Iran and Italy). Subjects were randomly assigned to either a placebo group, control group or intervention group to reduce allocation bias. Age of participants ranged from 18 to 65 years across eight included studies. Various regimens were examined in Table 1: Probiotics (genus *Lactobacillus*, *Streptococcus*, *Bifidobacterium*, *Enterococcus*), Prebiotics (Fructo-oligosaccharides [FOS], black bean, *Elaeagnus angustifolia L*., and some phytochemicals, like Berberine, silymarin, green tea essence) and synbiotics (a mixture of probiotics and prebiotics) at various doses. The study populations consisted of individuals at risk of thyroid disorders or with existing thyroid disease, encompassing the overweight/obese, GD patients, postmenopausal women, patients with hypothyroidism and DTC patients. The duration of intervention ranged from 4 weeks-12 months (Table 1).

**Table 1:** Characteristics of the studies included in this review.

Author, Year	Country	Sample size (n)	Age (Years)	Intervention Regimen	Control Regimen	Duration	Population	Findings	Jadad score and Study quality
**Probiotics/Prebiotics**									
Lin, 2022 [17]	China	39	18–65	Probiotics: *Bifidobacterium Tetravaccine* Tablets, Hangzhou Yuanda Biopharmaceutical Co., Ltd., SFDA approval number: S20060010; containing > 10^6^ CFU/tablet *B*. *infantis*, > 10^6^ CFU/tablet *L*. *acidophilus*, > 10^6^ CFU/tablet *E*. *faecalis*, > 10^5^ CFU/tablet *B*. *cereus* and > 10^6^ CFU/tablet total bacteria	Placebo (starch)	4 weeks	Post-thyroidectomy DTC patients	Probiotics reduce the incidence of complications in patients after THW	≥4, High quality
Huo, 2021 [16]	China	25	Not mentioned	Probiotics or Prebiotics: Group B: methimazole + black bean; Group C: Methimazole + probiotics (*Bifidobacterium longum*)	Methimazole treatment group	6 months	GD patients	MI+Black Bean reduced the FT3, FT4 and TSH at 1 month, while TRAb at month 6 was still higher than control; (2)MI+Probiotics reduced the TRAb in patients with GD.	<4, Low quality
Spaggiari, 2017 [33]	Italy	80	18–65	Probiotics: Multistrain probiotic supplement (*Bifidobacterium breve*, *Bifidobacterium longum*, *Bifidobacterium infantis*, *Lactobacillus acidophilus*, *Lactobacillus plantarum*, *Lactobacillus paracasei*, *Lactobacillus bulgaricus*, and *Streptococcus thermophilus*).	LT4	4 months	Patients with primary hypothyroidism (Caucasian)	Probiotic mixture does not alter directly LT4 therapy compensation in patients with primary hypothyroidism on substitutive treatment.	<4, Low quality
Han, 2022 [34]	China	18	Not mentioned	Prebiotics: Berberine treatment (Methimazole 20mg per tablet, 1 tablet each time once daily + Berberine tablets 0.1g per tablet, 3 tablets each time three times a day)	Methimazole treatment (20mg per tablet, 1 tablet each time once daily)	6 months	GD patients	Berberine restored the patient’ TSH and FT3 indices to normal levels, whereas MI alone restored only FT3.	<4, Low quality
Jalalvand, 2021 [35]	Iran	58	40–70	Prebiotics: *Elaeagnus angustifolia L*, 15g	Placebo (a combination of 7.5 g of corn starch and 7.5 g of isomalt)	10 weeks	Adult postmenopausal women	The increase in the TSH after *E*. *angustifolia* consumption was significant only based on within-group but not on the between-group analysis.	≥4, High quality
**Synbiotic**									
Kong, 2022 [36]	China: Taiwan	40	20–70	Synbiotic: *Lactobacillus johnsonii No*.*1088*, *Bacillus subtilis* (BS139), fermented sake lees, and green tea essence	Placebo capsules (microcrystalline cellulose, magnesium stearate and silicon dioxide)	12weeks	Overweight and obese subjects	TSH and T4 levels were significantly higher in the post-intervention supplementation group	≥4, High quality
Ramezani, 2023 [37]	Iran	51	25–55	Synbiotic: *Lactobacillus casei*, *Lactobacillus acidophilus*, *Lactobacillus rhamnus*, *Lactobacillus bulgaricus*, *Bifidobacterium Breve*, *Bifidobacterium Longum*, *Streptococcus thermophilus* plus FOS, 500mg, 109 CFU/g	Placebos contain lactose, magnesium stearate, talc, and silicon dioxide	10 weeks	Patients with hypothyroidism (BMI< 35)	TSH (p> 0.05) did not change significantly, FT4 significantly increased in both groups (p = 0.03 and p = 0.02 in symbiotic and placebo respectively).	≥4, High quality
Talebi, 2019 [19]	Iran	56	18–65	Synbiotic: seven freeze-dried probiotic strains, 7 × 10^9^ CFU *Lactobacillus Casei*, 2 × 10^9^ CFU *Lactobacillus Acidophilus*, 1.5 × 10^9^ CFU *Lactobacillus Rhamnosus*, 2 × 10^8^ CFU *Lactobacillus Bulgaricus*, 2 × 10^10^ CFU *Bifidobacterium Breve*, 7 × 10^9^ CFU *Bifidobacterium Longum*, 1.5 × 10^10^ CFU *Streptococcus Thermophilus*), FOS as a prebiotic, and lactose, magnesium stearate and talc as carrier substances, 500mg/d	Placebo capsules contained 375 mg starch, 22 mg lactose, 1 mg magnesium stearate, 1 mg silicon dioxide, and 1 mg talc.	8 weeks	Patients with hypothyroidism	TSH was significantly decreased after the 8-week intervention in the synbiotic group	≥4, High quality

Abbreviations: Randomised controlled trials; CFU: Colony-forming units; GD: Graves’ disease; DTC: Differentiated thyroid cancer; FOS: Fructo-oligosaccharide.

### 3.3 Subject characteristics

A total of 367 participants were included in the review, with 52.86% assigned to the intervention (probiotics, prebiotics, and/or synbiotics) group, 47.14% assigned to the control group. Probiotics or Prebiotics was commonly used treatments for thyroid disorders and other populations, constituting 61.34% and 58.38% of the intervention and control groups. Most studies focused on patients with hypothyroidism (48.45% and 53.76% in the treatment and control group, respectively), while hyperthyroidism was mainly caused by GD. Iran and China represented approximately half of the participants in both the intervention and control groups among the different study regions (Table 2).

**Table 2:** Demographic characteristics of participants.

Characteristics	Intervention	Placebo
**Total numbers, n (% total)**	194 (52.86%)	173 (47.14%)
**1. Type of intervention**		
Probiotics/Prebiotics	119 (61.34%)	101 (58.38%)
Synbiotics	75 (38.66%)	72 (41.62%)
**2. Population**		
2.1 Hypothyroidism and population at risk		
Hypothyroidism	94 (48.45%)	93 (53.76%)
DTC	23 (11.86%)	16 (9.25%)
Postmenopausal women	30 (15.46%)	28 (16.18%)
Obese	20 (10.31%)	20 (11.56%)
2.2 Hyperthyroidism and population at risk		
GD	27 (13.92%)	16 (9.25%)
**3. Nation**		
Iran	70 (36.08%)	52 (30.06%)
China	85 (43.81%)	80 (46.24%)
Italy	39 (20.10%)	41 (23.70%)

### 3.4 Risk of bias and study quality

**Fig 2:**
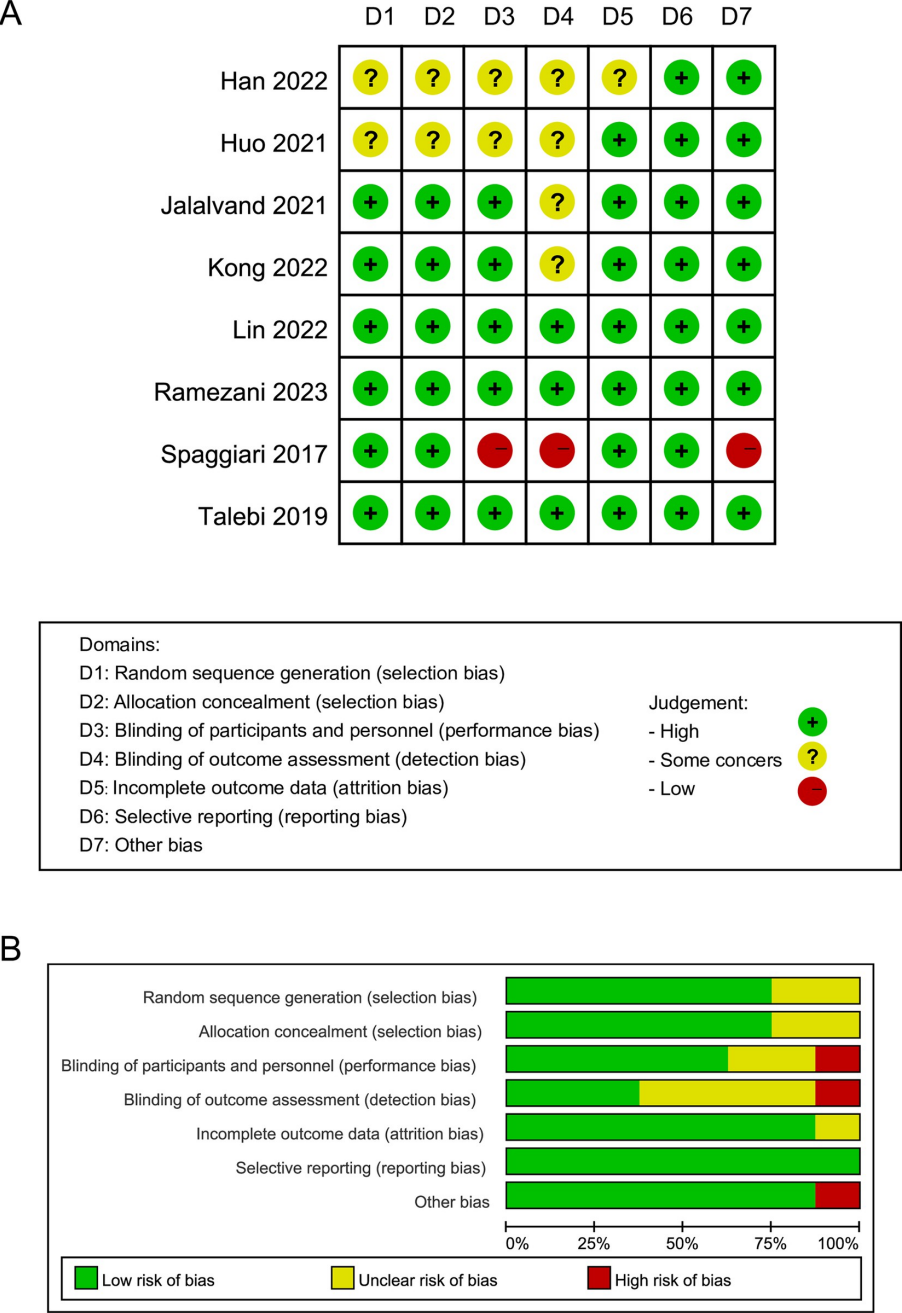
Risk of bias (ROB) analysis highlighting results in all domains examined within the nine identified studies (A) and overall risk of bias for included studies (B).

Out of the eight studies included in the analysis, one study was deemed to have a high risk of bias (ROB) overall, while four studies raised some concerns, and three studies were found to have low ROB across all domains (Fig 2A). The comprehensive overview of the ROB for the eight included studies is displayed in Fig 2B. Jadad scale was used for study quality assessment, resulting in five trials being classified as high quality (Jadad score ≥4), while the remaining trials were deemed low quality (Table 1).

### 3.5 Parameters of thyroid function

**Fig 3:**
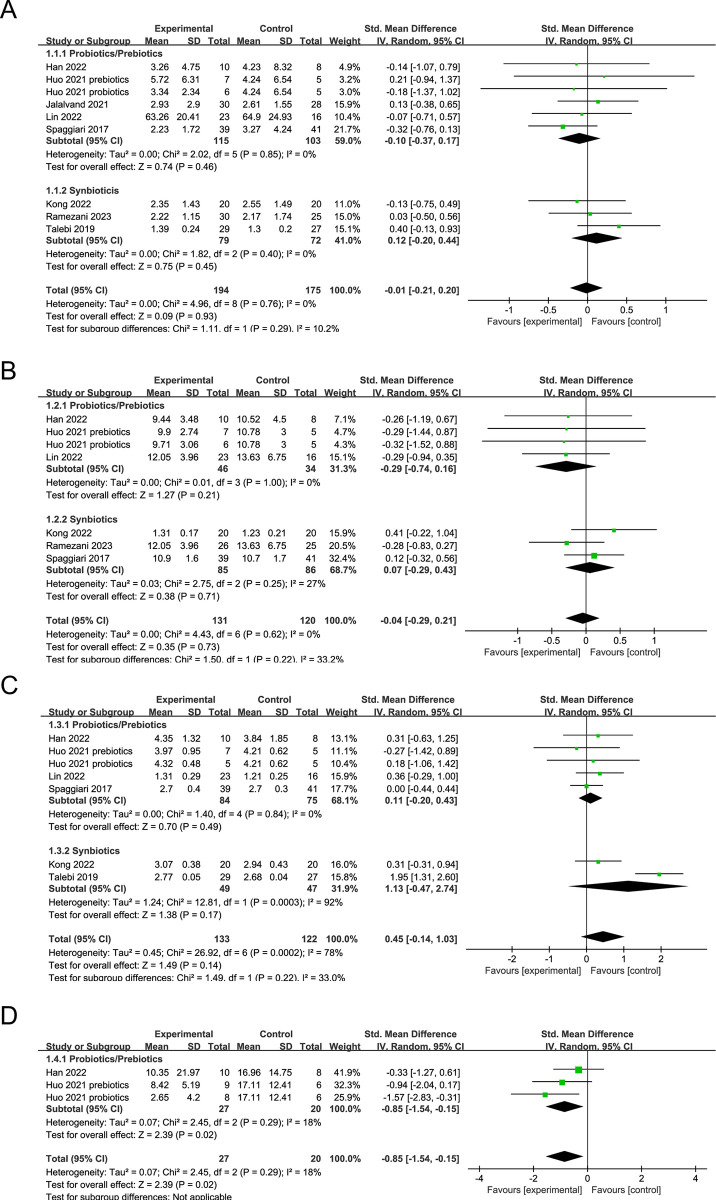
Forest plot displaying weighted mean difference and 95% confidence intervals for the impact on TSH (A), fT4 (B), fT3 (C) and TRAb (D) levels.

Meta-analysis of data from eight treatment arms did not reveal any significant effect of Probiotics/Prebiotics or Synbiotics on TSH (SMD: -0.01, 95% CI: −0.21, 0.20, *P* = 0.93; I^2^: 0.00%) (Fig 3A). Similarly, the results indicated that Probiotics/Prebiotics or Synbiotics intervention did not lead to an increase in fT4 (SMD: 0.04, 95% CI: −0.29, 0.21, *P* = 0.73; I^2^: 0.00%) or fT3 (SMD: 0.45, 95% CI: −0.14, 1.03, *P* = 0.14; I^2^: 78.00%) (Fig 3B and 3C). Subgroup analysis of probiotics/prebiotics and synbiotics treatment did not identify any significant changes in TSH, fT4, and fT3 levels between the intervention and control groups. However, there was a significant reduction in TRAb levels following probiotics/prebiotics treatment in GD patients (SMD: -0.85, 95% CI: -1.54, -0.15, *P* = 0.02; I^2^: 18.00%) (Fig 3D).

### 3.6 Publication bias

**Fig 4:**
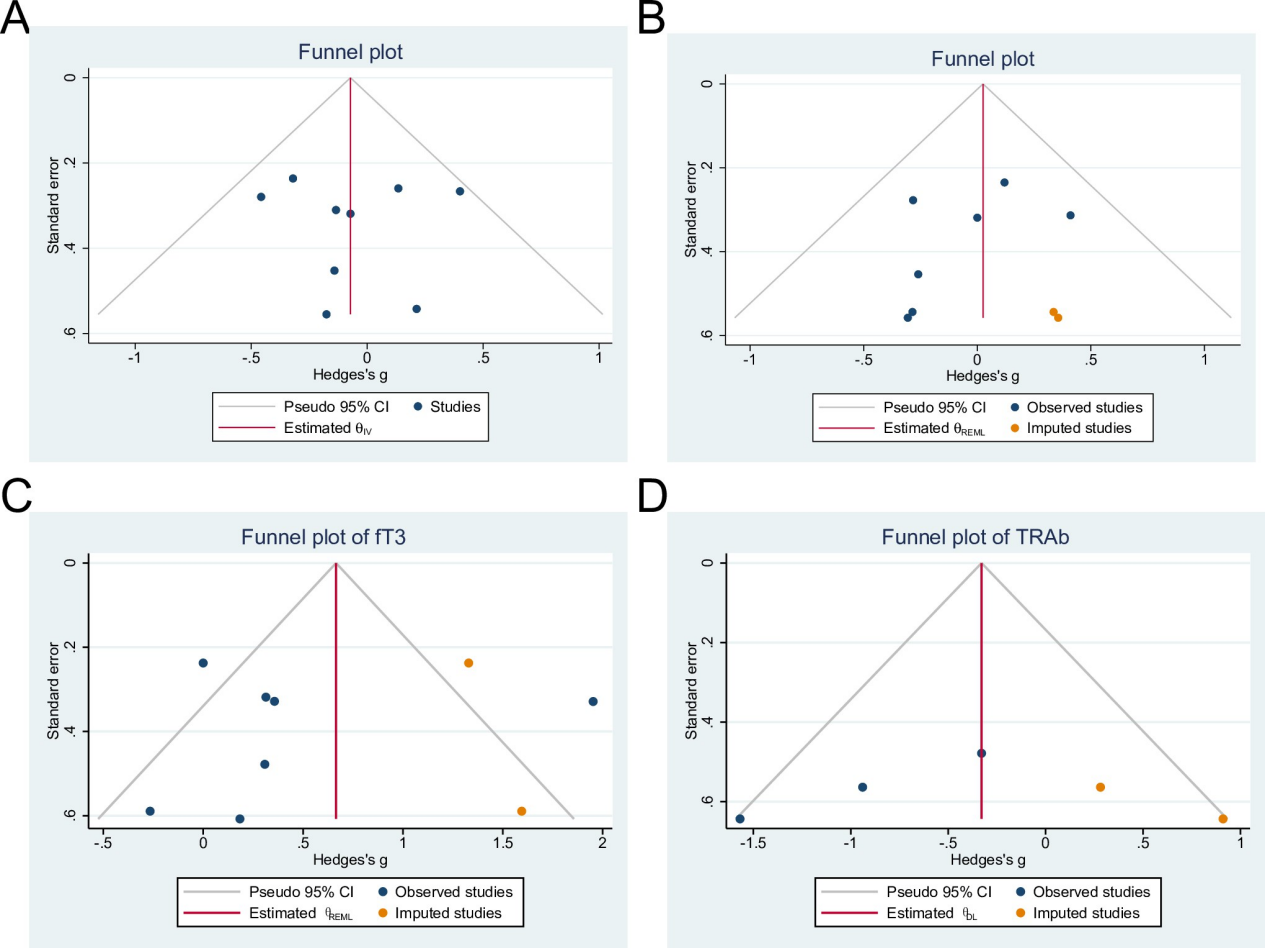
Funnel plot detailing publication bias in the studies reporting the impact on TSH (A), fT4 (B), fT3 (C) and TRAb (D) levels.

Visual inspection of funnel plots revealed asymmetry in the meta-analyses of TSH, fT4, fT3 and TRAb levels. There were 0, 2, 2 and 2 potentially imputed studies when using trim and fill correction of TSH, fT4, fT3 and TRAb (Fig 4 and Table 3). The results of the Egger’s regression tests and Begg’s rank correlation were summarized in Table 3. The results of sensitivity analysis demonstrated that the pooled effects of probiotics/prebiotics or synbiotics on serum concentrations of TSH, fT4, fT3 and TRAb were not changed after imputation using a correlation coefficient of 0.5.

**Table 3:** Imputed effect sizes and the results of Begg’s rank correlation and Egger’s regression tests for the meta-analysis on parameters of thyroid function. ^a^The number of imputed studies according to the trim and fill correction method; ^b^Begg’s rank correlation test; ^c^Egge’s weighted regression test.

	n^a^	SMD	95% CI	*P-value* ^b^	*P-value* ^c^
TSH	0	1.323734	-2.948813 to 3.311456	0.895	0.917
fT4	2	1.298914	-4.475367 to 2.202563	0.548	0.422
fT3	2	2.371505	-7.544722 to 4.647573	0.548	0.568
TRAb	2	4.774766	-68.14374 to 53.19457	0.296	0.362

## 4 Discussion

The purpose of this systematic review and meta-analysis was to investigate the efficacy of probiotics on thyroid function. eight studies involving 367 participants were deemed suitable for analysis. The findings indicate a significant decrease in TRAb levels among individuals with Graves’ disease who received probiotics/prebiotics supplementation, suggesting a potential therapeutic advantage in terms of TRAb. However, it should be noted that this clinical significance may not be significant. Additionally, no significant alterations were observed in TSH, fT4, and fT3 levels, which suggests that probiotics/prebiotics supplementation does not have an impact on thyroid function.

The human gut microbiome plays a crucial role in maintaining gastrointestinal homeostasis and may contribute to the development of thyroid disorders. Existing gut microbiota interventions such as probiotics, prebiotics, fecal microbiota transplantation, have been shown to be safe and beneficial for human health [26]. Several hypothesized mechanisms connect the microbiota to the development of thyroid diseases, including the generation of self-antigens through post-translational modification of proteins, activation of Toll-like receptor 4 by lipopolysaccharides, and the disruption of intercellular junctions in the intestine [27]. Moreover, the microbiome influences thyroid hormone levels by regulating iodine uptake, degradation, and enterohepatic circulation [28]. Additionally, minerals such as selenium, iron, and zinc, which are associated with thyroid function, significantly impact the interactions between the host and the gut microbiota [29]. A study found that the gut microbiota profile of thyroid cancer patients displayed a higher relative abundance of *Lactobacillaceae*, *Closteriaceae*, and *Enterobacteriaceae*, while *Prevotelaceae*, and *Bacillus* were less abundant compared to healthy adults [12]. However, fewer studies have investigated microbiota-targeted therapies for thyroid disease in humans, and sample size have typically been small. To gain a better understanding of the thyroid-gut axis and the potential for interventions, further well-designed human studies are needed.

Based on our meta-analysis data, we observed a significant decrease in TRAb levels in GD patients who underwent probiotics/prebiotics treatment in three treatment arms. TRAb is an important indicator of recovery in thyroid patients, as it reflects the presence of antibodies against the TSH receptor, a key immunological feature of GD [30]. Therefore, our findings suggest that probiotics or prebiotics may have potential benefits for GD patients. However, we did not observe any changes in TSH, fT4, and fT3 levels between the probiotics/prebiotics treatment group and the placebo group. This lack of correlation between antibody levels and the severity of GD or Hashimoto’s thyroiditis (HT) may explain why we did not observe any changes in these hormone levels. It is important to note that some probiotics have been associated with controversial effects on autoimmune thyroid disease. The genus *Lactobacillus* is generally considered a beneficial bacteria that contributes to host health through mechanisms such as immune regulation and maintenance of intestinal permeability. However, Moshkelgosha et al. found that a probiotic consortium called Lab4, which includes *Lactobacillus* and *Bifidobacterium*, increased the levels of orbital CD25+ Treg cells but promoted the development of GD/Graves’ orbitopathy phenotypes [31]. Additionally, higher abundance of *Lactobacillus* have been found in GD patients compared to healthy control [32]. These findings from animal models and GD patients indicate that *Lactobacillus* may not provide benefits to GD patients. Future studies should therefore focus on optimizing the probiotics formula by excluding Lactobacillus or exploring the use of synbiotics, a mixture of probiotics and prebiotics. It’s worth noting that no clinical trials have been conducted in GD patients using synbiotics.

Our study has some strengths. To our knowledge, this is the first meta-analysis to evaluate the impact of probiotics/prebiotics on thyroid function. We conducted robust subgroup analyses based on pre-defined criteria to provide detailed insights, and found moderate evidence supporting a decrease in TRAb levels in GD patients with probiotics/prebiotics supplementation. However, there are also several limitations to consider in this meta-analysis. Firstly, the pooled population included a limited number of subjects as some clinical trials had small sample sizes. Additionally, the duration of treatment in many trials was relatively short. Furthermore, due to heterogeneity and potential confounding factors like gender, it is challenging to precisely determine the effects. Another limitation is the variation in dosage of the interventions, particularly with probiotics. It is difficult to control and standardize the doses in microbiome-directed therapies. Additionally, it is important to note that all the studies demonstrating a significant reduction in TRAb were conducted by the Chen group in China, which may lead to publication bias, despite no significant publication bias reported here. Our findings provided valuable insights into the potential benefits of probiotics, prebiotics, and synbiotics on thyroid function, warranting further investigation. To strengthen the evidence, additional well-designed randomized controlled trials with larger sample sizes and standardized protocols are needed.

In conclusion, our meta-analysis indicates that supplementation with probiotics/prebiotics has no significant effect on thyroid hormone levels, while showing a modest decrease in TRAb levels.

## Supporting information

S1 Checklist PRISMA 2020 checklist. (DOCX)

S1 Table Search strategy for included studies. (DOCX)

S2 Table Data used in meta-analyses. (XLSX)

S1 Data (XLSX)
