# Supplementary material for: Effect of probiotics or prebiotics on thyroid function: A meta-analysis of eight randomized controlled trials
Source: PLoS One. 2024 Jan 11;19(1):e0296733. doi: 10.1371/journal.pone.0296733 (PMC10783727; doi:10.1371/journal.pone.0296733)
Supplement: S1 Table — (DOCX) [file pone.0296733.s002.docx]

Supplementary Table. Search strategy for included studies.

| MEDLINE via PubMed | #1 "Thyroid diseases"[All Fields] OR "Thyroid disorders"[All Fields] OR "Thyroid function"[All Fields] OR "hypothyroidism"[All Fields] OR "hyperthyroidism"[All Fields] OR "subclinical hypothyroidism"[All Fields] OR "subclinical hyperthyroidism"[All Fields] OR "thyroid cancer"[All Fields]  #2 "Prebiotics"[All Fields] OR "Probiotics"[All Fields] OR "synbiotics"[All Fields] OR "Yogurt"[All Fields] OR "milk"[All Fields] OR "Dairy product"[All Fields]  #1 AND #2  **Updated Search**  #3 Limited : clinical trial, and RCT |
| --- | --- |
| Scopus via Elsevier | #1TITLE-ABS-KEY(Thyroid diseases) OR TITLE-ABS-KEY(Thyroid disorders) OR TITLEABS-KEY(Thyroid function) OR TITLE-ABS-KEY(hypothyroidism) OR TITLE-ABS-KEY(hyperthyroidism) OR TITLE-ABS-KEY(subclinical hypothyroidism) OR TITLE-ABS-KEY(subclinical hyperthyroidism) OR TITLE-ABS-KEY(thyroid cancer)  #2 TITLE-ABS-KEY(Prebiotics) OR TITLE-ABS-KEY(Probiotics) OR TITLE-ABSKEY(synbiotics) OR TITLE-ABS-KEY(Yogurt) OR TITLE-ABS-KEY(milk) OR TITLEABS-KEY(Dairy product)  #1 AND #2  **Updated Search**  #3 TITLE-ABS-KEY (randomized AND controlled AND trial ) OR TITLE-ABS-KEY (randomized AND clinical AND trial ) OR TITLE-ABS-KEY (clinical AND trial ) OR TITLE-ABS-KEY (RCT) |
| Embase via Elsevier | #1 'Thyroid diseases' OR 'Thyroid disorders' OR 'Thyroid function' OR 'hypothyroidism'/exp OR hypothyroidism OR 'hyperthyroidism'/exp OR hyperthyroidism OR 'subclinical hypothyroidism' OR 'subclinical hyperthyroidism' OR 'thyroid cancer'  #2 'probiotics'/exp OR probiotics OR 'prebiotics'/exp OR prebiotics OR 'synbiotics'/exp OR synbiotics OR 'yogurt'/exp OR yogurt OR 'milk'/exp OR milk OR 'Dairy product '  #1 AND #2  **Updated Search**  #3 - 'crossover procedure':de OR 'double-blind procedure':de OR 'randomized controlled trial':de OR 'single-blind procedure':de OR random*:de,ab,ti OR factorial*:de,ab,ti OR crossover*:de,ab,ti OR ((cross NEXT/1 over*):de,ab,ti) OR placebo*:de,ab,ti OR ((doubl*NEAR/1 blind*):de,ab,ti) OR ((singl* NEAR/1 blind*):de,ab,ti) OR assign*:de,ab,ti OR allocat*:de,ab,ti OR volunteer*:de,ab,ti |
| Web of Science-Science Citation Index and Social Sciences Citation Index via Clarivate | #1 TS=(Thyroid diseases) OR TS=(Thyroid disorders) OR TS=(Thyroid function) OR TS=('hypothyroidism') OR TS=(hyperthyroidism) OR TS=(subclinical hypothyroidism) OR TS=(subclinical hyperthyroidism) OR TS=(thyroid cancer).  #2 TS=(probiotics) OR TS=(prebiotics) OR TS=(synbiotics) OR TS=(yogurt) OR TS=(milk) OR TS=(dairy product)  #1 AND #2  **Updated Search**  #3 Limited : TS=(randomized clinical trial) OR TS=(RCT) |
